# Toward FIAP^®^-Digital: an interpretable multimodal AI architecture for translational stratification and precision care in Autism

**DOI:** 10.3389/fninf.2026.1868068

**Published:** 2026-09-01

**Authors:** Yves Fuamba

**Affiliations:** Department of Autism Research, Antibiostress Clinics, Gloucester, ON, Canada

**Keywords:** artificial intelligence, autism spectrum disorder, explainable AI, multimodal integration, neuroinformatics, precision-stratified research, responsible AI, translational stratification

## Abstract

Autism spectrum disorder is characterized by substantial heterogeneity across biological burden, adaptive reserve, developmental timing, therapeutic engagement, and intervention responsiveness. This heterogeneity increasingly motivates the development of multimodal and artificial intelligence-supported approaches in autism research. However, many existing approaches emphasize diagnostic classification, symptom prediction, or generic outcome modeling, while fewer frameworks explicitly preserve clinically interpretable constructs linking biological burden, developmental state, therapeutic accessibility, and responsiveness to intervention. This Hypothesis and Theory article proposes FIAP^®^-Digital as a conceptual, hypothesis-generating, and human-supervised multimodal AI architecture for future translational stratification research in autism. FIAP^®^-Digital is designed to integrate multimodal inputs-including biological, physiological, developmental, contextual, therapeutic-process, and longitudinal indicators-into structured estimates of FIAP^®^ constructs such as the Biological Burden Index, energetic capacity, adaptive neurodevelopmental window accessibility, therapeutic engagement accessibility, neuroplastic accessibility, and higher-order translational profiles. The proposed architecture is construct-preserving rather than purely predictive. It emphasizes multimodal data organization, construct-level representation, temporal updating, uncertainty visibility, explainability, clinician-in-the-loop interpretation, and Responsible AI governance. The manuscript outlines candidate computational components, including multimodal fusion, temporal modeling, missing-data handling, uncertainty estimation, explainability mechanisms, and clinician-readable outputs. It also proposes operational computational representations of FIAP^®^ constructs and a stepwise human-supervised profiling workflow. Importantly, FIAP^®^-Digital is not presented as a validated diagnostic tool, treatment recommendation system, autonomous AI model, or clinically proven platform. The constructs described remain hypothetical and require empirical validation. The article therefore provides a neuroinformatics architecture and validation roadmap, including feasibility testing, construct validity, internal and external validation, longitudinal robustness, clinician usability, fairness assessment, and uncertainty calibration. Its primary contribution is to define a responsible computational pathway for testing whether FIAP^®^ constructs can support future precision-stratified autism research.

## Introduction

Autism spectrum disorder (ASD) is marked by substantial heterogeneity across biology, development, and therapeutic responsiveness (Lai et al., 2014; Lord et al., 2020; Lombardo et al., 2019; American Psychiatric Association, 2022; Happé and Frith, 2020). Within the broader FIAP^®^ ecosystem, this heterogeneity has been conceptualized through interacting translational layers, including multidomain biological burden, adaptive reserve, developmental accessibility, therapeutic engagement, and neuroplastic relevance. Earlier FIAP^®^ papers argued that therapeutic benefit is not equally accessible across autistic individuals because interventions are received by neurodevelopmental systems operating under different conditions of burden, reserve, timing, and functional usability.

Autism heterogeneity is increasingly studied through genetic, diagnostic, phenotypic, biomarker, and digital-health approaches. However, few frameworks integrate biological burden, adaptive reserve, therapeutic engagement, developmental timing, and intervention responsiveness into a construct-preserving computational architecture. This gap is especially relevant to neuroinformatics, where multimodal data can improve measurement only if computational representations remain interpretable, testable, and aligned with clearly defined constructs.

The present paper asks what type of computational architecture would eventually be required if such a framework were to be digitally integrated in a scientifically and clinically meaningful way. FIAP^®^ is not a single-marker model, nor a simple correlational framework. It is multidomain, dynamic, developmental, and profile-based. Its clinically relevant meaning emerges from the interaction of several inferential layers rather than from raw signals alone. As a result, any future digital realization of FIAP^®^ must do more than aggregate multimodal information. It must preserve the logic of the constructs from which the ecosystem is built.

This distinguishes the present paper from the companion clinical-translational FIAP^®^-Digital roadmap. That paper emphasized why FIAP^®^-Digital matters for therapeutic accessibility, individualized intervention alignment, and future precision-oriented research in autism. The present paper emphasizes how a digital system could be built to support those goals. Its main concerns are multimodal integration, construct estimation, profile inference, explainability, clinician-in-the-loop design, and staged validation logic.

A key premise of this manuscript is that a useful digital framework in autism should be construct-preserving rather than construct-erasing. Many AI systems operate by optimizing prediction from raw data without preserving human-interpretable inferential layers. That may be appropriate in some tasks, but it is poorly suited to the FIAP^®^ ecosystem. The purpose of FIAP^®^-Digital is not merely to predict a label or outcome. It is to support translational stratification in a way that remains clinically interpretable, uncertainty-aware, and open to empirical validation (Amann et al., 2020; Kelly et al., 2019; Rajkomar et al., 2019).

The objective of this paper is to define the architecture that such a system would require. We first explain the research gap and novelty of FIAP^®^-Digital. We then define its intended context of use and boundaries. Next, we describe why autism translational heterogeneity requires multimodal AI integration and outline the proposed neuroinformatics architecture, including data quality control, multimodal fusion, temporal modeling, missing-data handling, uncertainty estimation, explainability, and clinician-in-the-loop review. We then operationalize FIAP^®^ constructs computationally, propose a human-supervised profiling workflow, and outline a validation roadmap, implementation challenges, and Responsible AI governance requirements.

**Figure 1:**
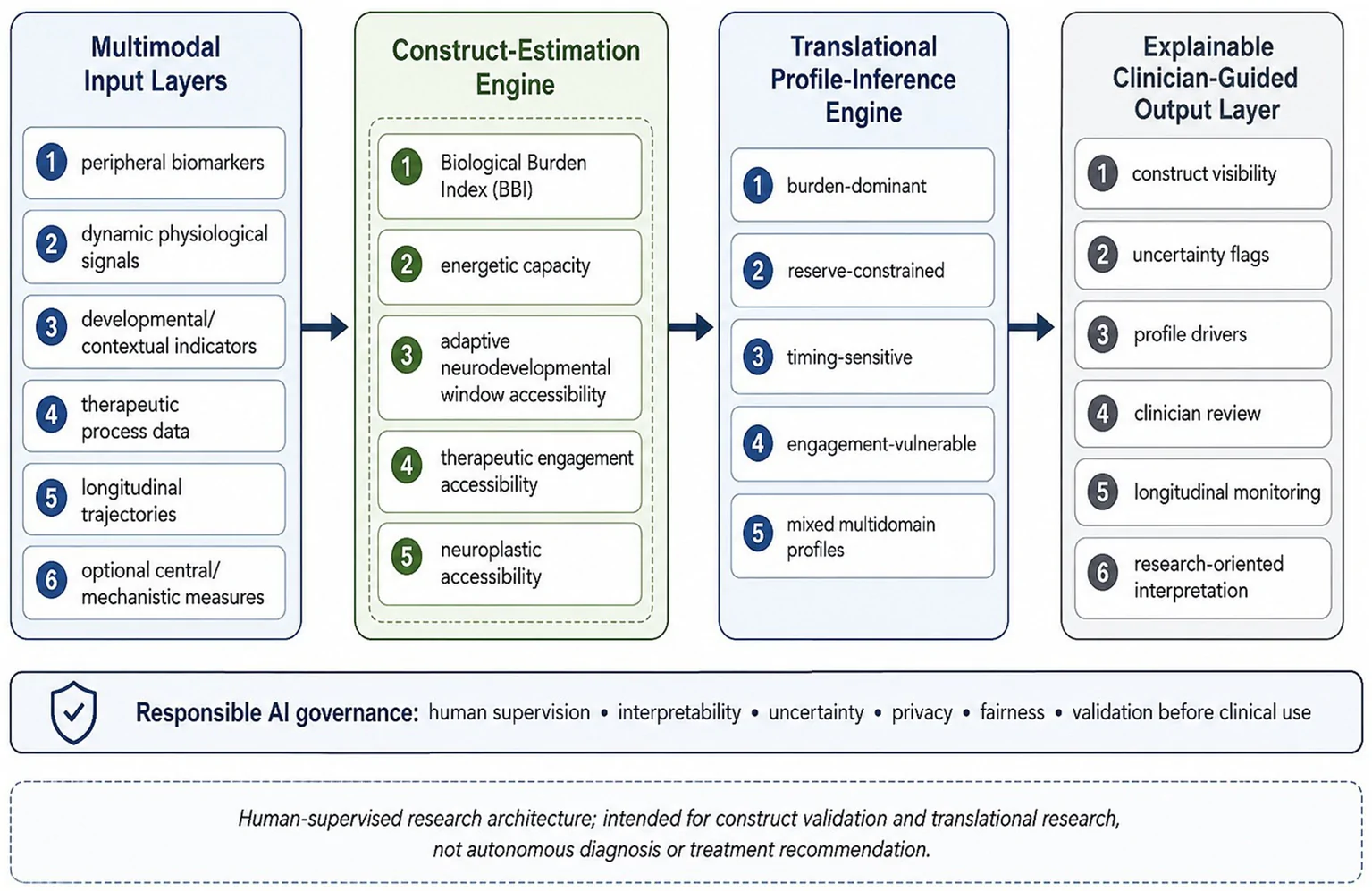
Interpretable multimodal AI architecture of FIAP^®^-Digital for translational stratification and precision care in autism. Multimodal input layers include peripheral biomarkers, dynamic physiological signals, developmental/contextual information, therapeutic process data, longitudinal trajectories, and, where available, central mechanistic measures. These inputs feed a construct-estimation engine that generates structured estimates of multidomain biological burden, energetic capacity, adaptive neurodevelopmental window accessibility, therapeutic engagement accessibility, and neuroplastic accessibility. A translational profile-inference engine then identifies higher-order multidomain configurations such as burden-dominant, reserve-constrained, timing-sensitive, engagement-vulnerable, and mixed profiles. Finally, an explainable clinician-guided output layer supports research-oriented interpretation, individualized hypothesis generation, and longitudinal monitoring pending validation.

Figure 1 summarizes the overall FIAP^®^-Digital architecture, and Table 1 summarizes the main computational layers and their translational functions.

**Table 1:** Computational layers of FIAP^®^-Digital and their translational functions.

Computational layer	Primary role	Examples of internal targets	Translational function
Multimodal input architecture	Organize heterogeneous data streams by modality and temporal structure	Biomarkers, physiology, developmental/contextual variables, therapeutic process data	Preserves meaningful multimodal substrate for inference
Construct-estimation engine	Infer core FIAP^®^ constructs from multimodal inputs	BBI, energetic capacity, window accessibility, engagement accessibility, neuroplastic accessibility	Converts raw data into interpretable translational layers
Translational profile-inference engine	Infer higher-order multidomain configurations from construct interaction	Burden-dominant, reserve-constrained, timing-sensitive, engagement-vulnerable, mixed profiles	Makes heterogeneity computationally visible and testable
Explainability and clinician-in-the-loop layer	Expose reasoning pathways, uncertainty, and review structure	Construct visibility, profile drivers, uncertainty flags	Supports trustworthy clinician-guided research interpretation
Precision-oriented output layer	Translate profiles into individualized interpretive support pending validation	Timing hypotheses, reserve-support priorities, burden-related interpretation	Supports future precision-oriented research and therapeutic reasoning
Longitudinal updating layer	Track accessibility change across time	Profile shifts, stability trends, timing changes, reserve fluctuation	Enables dynamic monitoring and repeated therapeutic alignment hypotheses

## Research gap and novelty of FIAP^®^-digital

Recent advances in autism research increasingly recognize that autism heterogeneity cannot be adequately captured by diagnostic status alone. Large-scale studies have examined heterogeneity across genetic architecture, diagnostic timing, sex, intellectual disability, developmental profiles, behavioral phenotypes, biomarkers, and response trajectories. In parallel, machine learning and multimodal AI approaches have been applied to autism-related classification, biomarker discovery, behavioral analysis, symptom prediction, and digital phenotyping. These developments support the view that autism is not a single homogeneous clinical entity, but a multidimensional neurodevelopmental condition requiring stratified and biologically informed research approaches.

Despite these advances, several gaps remain. First, many AI approaches in autism are optimized for diagnostic classification or prediction of broad clinical labels rather than for construct-preserving translational interpretation. Second, multimodal data are often fused into statistical representations that may improve performance but reduce visibility of the intermediate clinical and biological constructs that matter for interpretation. Third, few computational frameworks explicitly connect biological burden, adaptive reserve, developmental timing, therapeutic engagement, neuroplastic accessibility, and intervention responsiveness. Fourth, many digital health approaches do not sufficiently distinguish research-oriented hypothesis generation from clinically validated decision support.

This gap is not a rejection of existing multimodal AI research. Rather, it builds on prior work showing that multimodal machine learning can integrate heterogeneous signal classes, that healthcare AI requires careful translation, evaluation, and workflow integration, and that EHR-based and digital data can support representation learning while also introducing bias, missingness, and interpretability challenges (Baltrušaitis et al., 2019; Kelly et al., 2019; Rajkomar et al., 2019; Shickel et al., 2018; Esteva et al., 2019). In autism specifically, prior biomarker, phenotyping, and machine-learning studies have helped decompose heterogeneity, but most have not been designed to preserve intermediate constructs linking biological burden, adaptive reserve, developmental timing, therapeutic engagement accessibility, and responsiveness to intervention (Lombardo et al., 2019; Loth et al., 2023; Beversdorf et al., 2023; Elbattah, 2024; Malik et al., 2025). The proposed research gap is therefore architectural: FIAP^®^-Digital asks how multimodal data can be organized so that clinically meaningful constructs remain visible, testable, uncertainty-aware, and open to human review.

FIAP^®^-Digital is proposed to address these gaps by providing a construct-preserving and human-supervised neuroinformatics architecture. Its novelty lies not in claiming a new machine learning algorithm or validated clinical tool, but in organizing multimodal autism data around a defined set of translational constructs within the FIAP^®^ ecosystem. These constructs include the Biological Burden Index, energetic capacity, adaptive neurodevelopmental window accessibility, therapeutic engagement accessibility, neuroplastic accessibility, and higher-order translational profiles.

In contrast to models focused primarily on diagnostic classification, FIAP^®^-Digital aims to support hypothesis testing about why autistic individuals may differ in therapeutic accessibility and intervention responsiveness. In contrast to black-box prediction pipelines, it emphasizes construct-level visibility, uncertainty estimation, clinician review, longitudinal updating, and Responsible AI governance. In contrast to single-domain stratification models, it proposes a layered architecture in which biological, physiological, developmental, contextual, therapeutic-process, and longitudinal indicators contribute to interpretable translational profiles.

## Direct comparison with existing AI and multimodal autism frameworks

A more direct comparison further clarifies the proposed novelty. Existing AI and multimodal autism frameworks often prioritize diagnostic classification, risk prediction, behavioral phenotyping, biomarker discovery, or outcome prediction. By contrast, FIAP^®^-Digital is designed as a construct-guided translational architecture: multimodal inputs are mapped first to interpretable FIAP^®^ constructs, then to uncertainty-aware translational profiles, and only then to research-oriented hypotheses about therapeutic accessibility and intervention responsiveness. In this sense, the architecture differs from generic multimodal precision psychiatry pipelines along six dimensions: (1) its primary goal is construct-guided stratification rather than diagnostic classification; (2) its intermediate representations are explicitly defined constructs rather than opaque latent variables; (3) its outputs are clinician-readable research profiles rather than autonomous decisions; (4) its explainability is built around construct contributions, missingness, and uncertainty rather than *post-hoc* feature importance alone; (5) its validation roadmap includes construct validity, usability, fairness, and uncertainty calibration rather than model performance alone; and (6) its current context of use is hypothesis generation and prototype testing, not clinical decision support.

Accordingly, FIAP^®^-Digital should be read as a construct-preserving architecture positioned between three bodies of work: multimodal machine learning, AI in healthcare, and autism stratification research. Multimodal machine learning provides the technical rationale for combining heterogeneous data streams; healthcare AI literature emphasizes evaluation, calibration, workflow integration, and safety; and autism precision research emphasizes heterogeneity, biomarker limitations, and the need for stratified approaches (Baltrušaitis et al., 2019; Topol, 2019; Kelly et al., 2019; Loth et al., 2023). FIAP^®^-Digital does not compete with these frameworks as a new algorithm. It proposes a clinically interpretable mapping layer through which multimodal indicators can be transformed into explicit, reviewable, and empirically testable FIAP^®^ constructs.

The proposed contribution is therefore architectural and methodological: FIAP^®^-Digital defines a pathway through which FIAP^®^ constructs could be operationalized computationally, tested empirically, and refined before any clinical use is considered. Its intended role is to guide future feasibility studies, prototype development, construct validation, and precision-stratified autism research rather than to provide immediate diagnostic or therapeutic decision-making.

## Intended context of use and boundaries

FIAP^®^-Digital should be understood as a research-oriented, hypothesis-generating architecture intended to support future empirical validation of FIAP^®^ constructs. Its proposed context of use is translational autism research, not immediate clinical deployment. The architecture is intended to help organize multimodal data, generate construct-level estimates, support longitudinal profiling, and produce clinician-readable translational profiles for research interpretation and hypothesis testing.

In its current form, FIAP^®^-Digital is not a diagnostic tool, treatment protocol, autonomous AI system, validated predictive model, or clinical decision-support platform. It should not be used to diagnose autism, assign treatment, determine service eligibility, predict individual outcomes, or replace clinical judgment. The framework has not yet undergone construct validation, prototype testing, prospective validation, external replication, or clinical utility assessment. No evidence of clinical effectiveness is claimed.

The appropriate early context of use is therefore limited to research settings in which data collection, participant protection, ethical oversight, clinician review, and methodological transparency are explicitly maintained. In such settings, FIAP^®^-Digital may help structure pilot studies by organizing clinically accessible indicators into research profiles, documenting missingness and uncertainty, and generating hypotheses about relationships among biological burden, adaptive reserve, developmental timing, therapeutic engagement, neuroplastic accessibility, and intervention responsiveness.

This boundary is important for both scientific and ethical reasons. Scientifically, unvalidated constructs should not be treated as established measurement instruments. Ethically, AI-supported outputs in autism research must avoid deterministic labeling, premature clinical interpretation, and inequitable use in service allocation. FIAP^®^-Digital is therefore positioned as a staged architecture: concept definition, operational representation, feasibility testing, construct validation, prototype development, internal and external validation, Responsible AI evaluation, and only then potential future clinical translation.

## Why autism translational heterogeneity requires multimodal AI integration

Autism translational heterogeneity requires multimodal AI integration because the relevant variability is not confined to one domain, one timescale, or one inferential level. The FIAP^®^ ecosystem proposes that therapeutic accessibility in autism is shaped by the interaction of multidomain biological burden, adaptive reserve, developmental timing, therapeutic engagement, and neuroplastic accessibility. This means that no single signal class is likely to capture the clinically meaningful structure of heterogeneity on its own.

Relevant information may emerge from peripheral biomarkers, autonomic and physiological dynamics, developmental status, contextual conditions, therapeutic process measures, and possibly central nervous system-related indicators. These signal classes do not contribute identical information. Some are more informative for burden, others for reserve, others for timing accessibility, and others for therapeutic usability (Jin and Wang, 2024; Loth et al., 2023).

A computational framework is therefore needed not merely because the data are large, but because the heterogeneity is distributed, layered, dynamic, and interaction-dependent. Multimodal integration is particularly appropriate because the FIAP^®^ framework is construct-dependent rather than raw-signal dependent. Its goal is to estimate interpretable translational layers and higher-order profiles from several distinct but interacting modalities (Elbattah, 2024; Malik et al., 2025).

However, multimodal integration also increases methodological risk. Data may be incomplete, noisy, context-dependent, temporally misaligned, or differentially available across populations and settings. For this reason, FIAP^®^-Digital must be designed as a missingness-tolerant, uncertainty-aware, and human-supervised architecture rather than as a fully automated prediction pipeline.

## Computational targets: FIAP^®^ constructs

The computational value of FIAP^®^-Digital depends on what the system is actually trying to estimate. A useful architecture should be optimized for construct-preserving inference rather than raw prediction alone (Chen et al., 2022; Beversdorf et al., 2023; Loth et al., 2023).

Its primary computational targets are the core FIAP^®^ constructs: the Biological Burden Index (BBI), energetic capacity, adaptive neurodevelopmental window accessibility, therapeutic engagement accessibility, neuroplastic accessibility, and higher-order translational profiles. BBI would function as an upstream latent construct inferred from multiple biological and physiological signal classes. Energetic capacity would function as a distinct reserve layer, likely informed by dynamic and repeated measures (Mahony and O'Ryan, 2022; Frye et al., 2024).

Adaptive neurodevelopmental window accessibility would require integration of developmental phase, context, burden, and reserve. Therapeutic engagement accessibility would require therapeutic process-linked signals. Neuroplastic accessibility would function as an integrative downstream construct. Longitudinal updating and uncertainty visibility are also core computational targets because the FIAP^®^ ecosystem is dynamic and non-deterministic by design.

To make the FIAP^®^ constructs computationally testable, each construct must be translated into candidate input domains, measurable indicators, computational representations, and interpretable outputs. Table 2 provides an initial operational representation. These representations are not proposed as validated measures; rather, they define a starting point for future construct validation, feasibility testing, and empirical refinement.

**Table 2:** Operational computational representation of FIAP^®^ constructs.

FIAP^®^ construct	Conceptual definition	Candidate input domains	Measurable indicators	Computational representation	Expected output
Biological Burden Index (BBI)	Multidomain biological and physiological burden potentially constraining regulation, adaptive reserve, and therapeutic accessibility	Sleep, GI symptoms, immune/allergic burden, fatigue, pain, nutrition, sensory-physiological stress, autonomic regulation, metabolic or mitochondrial indicators where available	Sleep disruption, GI symptom frequency, fatigue ratings, pain/discomfort indicators, sensory overload, stress recovery difficulty, autonomic variability, relevant biomarkers where feasible	Composite burden profile; multidomain burden vector; latent burden estimate; domain-specific burden subscores with uncertainty flags	Burden-dominant profile; burden domains requiring further research review; burden-related uncertainty estimate
Energetic capacity	Adaptive reserve available for engagement, learning, recovery, and sustained participation	Fatigue, endurance, sleep quality, recovery patterns, activity tolerance, physiological reserve, caregiver/clinician reports of depletion	Fatigue frequency, endurance during tasks, recovery time, sleep adequacy, variability in participation, state-dependent shutdown or withdrawal indicators	Reserve constraint index; dynamic reserve profile; longitudinal fluctuation estimate	Reserve-constrained profile; low-reserve periods; candidate timing constraints for research interpretation
Adaptive neurodevelopmental window accessibility	Degree to which developmental timing, regulation, context, and biological state permit access to learning or therapeutic benefit	Developmental stage, contextual demands, environmental stability, regulation, burden, reserve, transition periods, therapeutic timing	State readiness, contextual stress, transition sensitivity, learning availability, variability across settings, timing-related changes in engagement	Dynamic window accessibility estimate; state-sensitive readiness profile; context-conditioned accessibility score	Timing-sensitive profile; periods of increased or reduced accessibility; longitudinal readiness pattern
Therapeutic engagement accessibility / TEI	Functional availability to participate in and benefit from therapeutic or developmental support	Regulation, attention, interactional access, responsiveness to support, persistence, adaptive effort, contextual transfer, relational-motivational engagement	Attentional availability, tolerance of interaction, persistence during challenge, response to prompting, generalization, adaptive effort, relational engagement	Engagement accessibility profile; TEI domain vector; engagement-vulnerability estimate; session-to-session change profile	Engagement-vulnerable profile; domain-specific engagement barriers; longitudinal engagement trajectory
Neuroplastic accessibility	Estimated capacity for adaptive change when developmental, biological, contextual, and therapeutic conditions are favorable	Learning response, adaptive flexibility, developmental gains, intervention responsiveness, burden/reserve balance, engagement availability	Response to support, learning gains, flexibility, generalization, developmental change, reduction in barriers, stability of gains	Integrative plasticity-accessibility estimate; longitudinal change signal; responsiveness trajectory	Plasticity-accessible or plasticity-constrained profile; candidate responsiveness pattern requiring empirical validation
Translational profile configuration	Higher-order multidomain pattern inferred from interactions among FIAP^®^ constructs	BBI, energetic capacity, window accessibility, TEI, neuroplastic accessibility, contextual variables, longitudinal change	Construct estimates, construct interactions, uncertainty estimates, profile stability, temporal shifts	Probabilistic multidomain profile; mixed-profile configuration; dynamic stratification pattern	Burden-dominant, reserve-constrained, timing-sensitive, engagement-vulnerable, plasticity-constrained, or mixed profile

This table is intended to make the proposed constructs computationally explicit by linking each construct to candidate input domains, measurable indicators, computational representations, and expected outputs.

## Proposed FIAP^®^-Digital neuroinformatics architecture

### Multimodal input layers

The first architectural layer consists of multimodal data inputs. These inputs may include biological indicators, physiological dynamics, developmental and contextual variables, therapeutic process indicators, longitudinal trajectory data, and, where available, central or mechanistic neurodevelopmental measures. Biological indicators may include immune, inflammatory, metabolic, mitochondrial, oxidative stress, gastrointestinal, sleep-related, or stress-related variables. Physiological indicators may include autonomic regulation, recovery patterns, fatigue-related signals, arousal variability, and other state-sensitive measures. Developmental-contextual inputs may include developmental stage, communication profile, adaptive functioning, environmental demands, family context, school context, and intervention history. Therapeutic process inputs may include tolerance, persistence, responsiveness to support, generalization, engagement availability, and session-to-session variability.

These modalities are not treated as interchangeable data streams. In FIAP^®^-Digital, each modality is organized according to its potential relevance to specific FIAP^®^ constructs. For example, sleep, gastrointestinal symptoms, fatigue, pain, and physiological stress may contribute primarily to Biological Burden Index estimation, while persistence, regulatory readiness, attentional access, and responsiveness to guidance may contribute more directly to therapeutic engagement accessibility. This modality-to-construct mapping is intended to prevent premature fusion of heterogeneous inputs into uninterpretable latent representations.

### Data preprocessing and quality control

Before any construct-level inference, FIAP^®^-Digital requires a structured preprocessing and quality-control layer. This layer documents the source, timing, reliability, completeness, and clinical relevance of each data element. Each variable should be accompanied by metadata describing whether it was obtained through caregiver report, clinician observation, structured assessment, device-based measurement, record review, or longitudinal monitoring. Data elements should also be tagged for timestamp, context, missingness, uncertainty, and measurement quality.

This quality-control step is central because autism-related data are often incomplete, context-dependent, and unevenly available across families, clinics, schools, and research settings. A FIAP^®^-Digital workflow should therefore avoid treating all data elements as equally reliable or equally available. Instead, the architecture should preserve data provenance and explicitly represent data quality as part of the output.

### Multimodal fusion strategies

Several candidate multimodal fusion strategies may be relevant to FIAP^®^-Digital. Early fusion combines variables from different modalities before modeling. Intermediate fusion combines modality-specific representations at an intermediate latent or construct level. Late fusion combines outputs generated separately from different modalities. A hybrid fusion strategy may combine all three approaches depending on the construct being estimated.

For FIAP^®^-Digital, intermediate or construct-level fusion is likely the most appropriate starting point because it preserves interpretability. Rather than merging all data into a single undifferentiated feature space, modality-specific indicators can first be mapped to candidate constructs. For example, sleep disruption, gastrointestinal burden, fatigue, and stress reactivity may be integrated into a BBI-related representation, while regulation, attention, persistence, and responsiveness to support may be integrated into a therapeutic engagement representation. Higher-order translational profiles can then be inferred from interactions among construct-level estimates.

This construct-level fusion strategy distinguishes FIAP^®^-Digital from purely performance-driven multimodal systems. The objective is not only predictive accuracy, but preservation of translational meaning.

### Temporal modeling and longitudinal updating

Autism intervention responsiveness is dynamic. Biological burden, energetic reserve, engagement availability, contextual demands, and developmental readiness may vary across time. FIAP^®^-Digital therefore requires temporal modeling rather than static one-time classification.

Candidate temporal approaches may include longitudinal mixed-effects models, state-space models, dynamic Bayesian updating, repeated-measures trajectory models, temporal latent profile models, recurrent architectures, or transformer-based longitudinal representations. In early feasibility phases, simpler longitudinal tracking and rule-based temporal summaries may be more appropriate than complex deep learning models. The architecture should allow gradual methodological escalation as data volume and validation maturity increase.

Temporal modeling would allow FIAP^®^-Digital to represent whether a profile is stable, fluctuating, improving, worsening, or context-sensitive. This is especially important for constructs such as adaptive neurodevelopmental window accessibility, therapeutic engagement accessibility, and energetic capacity, which may change with sleep, stress, illness, therapeutic environment, developmental transition, or intervention intensity.

### Missing-data handling

Missing data are expected in autism research and clinical contexts. In FIAP^®^-Digital, missingness should not be treated only as a technical nuisance. It may also carry interpretive meaning, especially when certain modalities are systematically unavailable because of service access, family burden, sensory intolerance, cost, geographic location, or institutional constraints.

The architecture should therefore include modality-specific missingness flags, data-completeness scores, and uncertainty inflation when key information is unavailable. Imputation may be used cautiously in later validated models, but early-stage FIAP^®^-Digital development should prioritize transparency over forced completion. When data are insufficient to estimate a construct reliably, the system should explicitly indicate low confidence rather than generating a deterministic profile.

### Uncertainty estimation

Uncertainty estimation is a core requirement of FIAP^®^-Digital. Because FIAP^®^ constructs are not yet validated measures, and because multimodal autism data may be noisy, incomplete, and context-dependent, outputs should be probabilistic and confidence-aware. Candidate approaches may include confidence intervals, calibrated probabilities, Bayesian posterior estimates, ensemble uncertainty, data-completeness weighting, sensitivity analyses, and uncertainty flags.

Uncertainty should be visible at multiple levels: variable level, construct level, profile level, and longitudinal interpretation level. For example, the system may estimate a burden-dominant profile with high confidence when multiple convergent indicators are present, but may estimate the same profile with low confidence when key biological or contextual data are missing. This uncertainty visibility is essential for ethical use, clinician review, and avoidance of overinterpretation.

### Explainability and human supervision

Explainability in FIAP^®^-Digital should be construct-centered rather than purely feature-centered. A clinician or researcher should be able to inspect which data domains contributed to each construct estimate, which constructs contributed to each profile, and how uncertainty affected the interpretation. Explanations should include profile drivers, missing-data warnings, data-quality notes, and construct-level summaries.

Human supervision is required at every stage. Clinicians and qualified researchers should be able to review, contextualize, contest, and revise interpretations. The system should not generate autonomous diagnostic, prognostic, or treatment recommendations. Instead, it should produce structured, transparent, and reviewable outputs that support hypothesis generation, research stratification, and future empirical validation.

**Figure 2:**
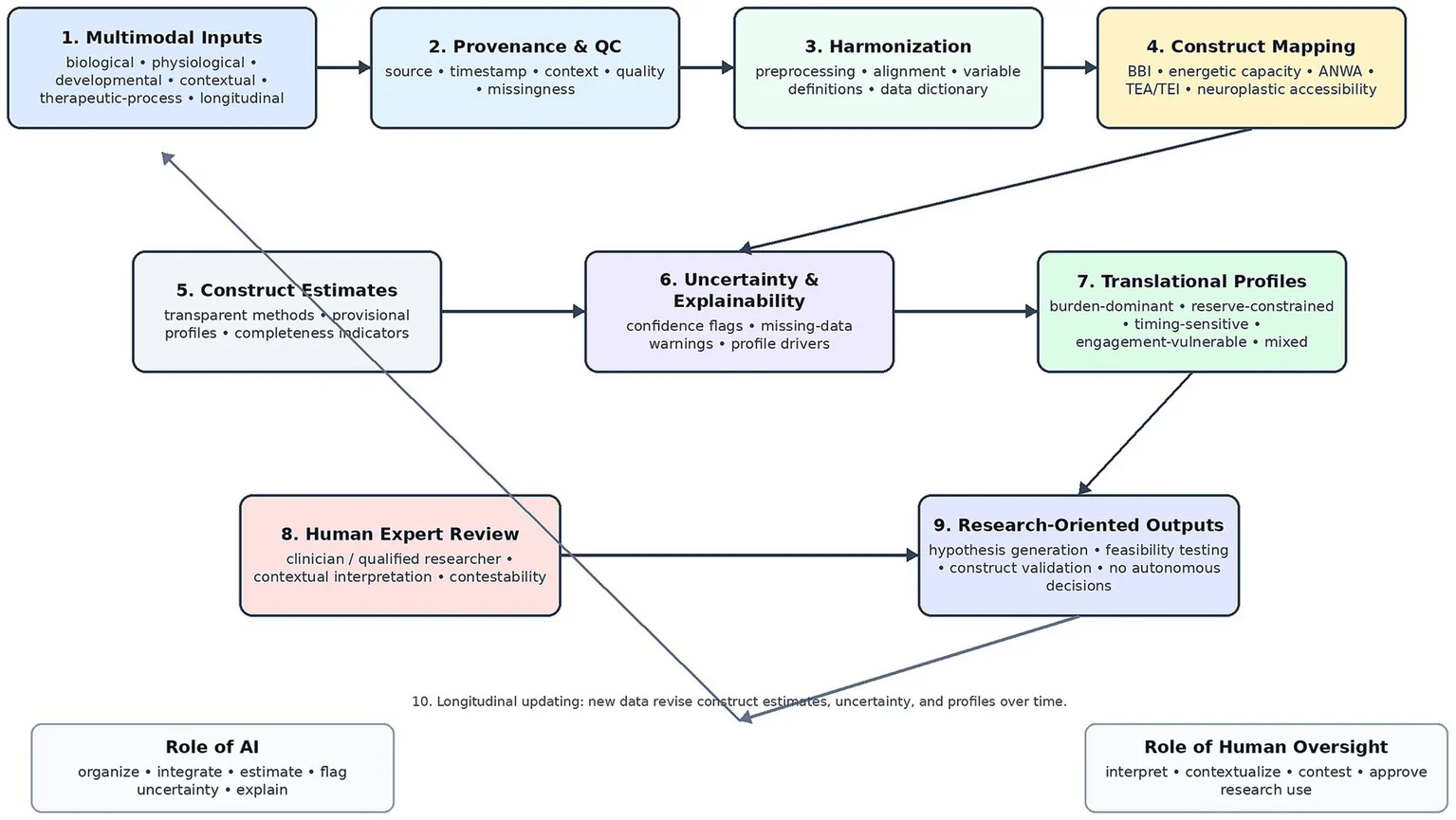
Human-supervised multimodal data flow and FIAP^®^-Digital profiling workflow. The figure illustrates the proposed multimodal data flow from available biological, physiological, developmental, contextual, therapeutic-process, and longitudinal indicators to research-oriented translational profiling. Data are first documented with source, timestamp, context, quality, provenance, and missingness information. Indicators are then harmonized by modality and mapped to candidate FIAP^®^ constructs, including BBI, energetic capacity, adaptive neurodevelopmental window accessibility, therapeutic engagement accessibility, and neuroplastic accessibility. The AI layer supports multimodal organization, construct-level estimation, missing-data detection, uncertainty flagging, longitudinal pattern recognition, and clinician-readable explanation. Outputs are then reviewed by clinicians or qualified researchers for contextual interpretation, contestability, and revision. Profiles are updated longitudinally as new data become available. The workflow is intended for research stratification, hypothesis generation, feasibility testing, and construct validation, not for autonomous diagnosis, treatment recommendation, service allocation, or clinical decision-making.

The proposed architecture therefore functions as a staged interpretive pipeline: multimodal inputs are documented and quality-controlled, mapped to FIAP^®^ constructs, integrated through construct-preserving fusion, updated longitudinally, accompanied by uncertainty estimates, and reviewed by human experts before any research interpretation is made. This working principle is summarized in Algorithm 1 and visually represented in Figure 2, which now shows the flow of multimodal data from input sources through quality control, harmonization, construct mapping, uncertainty-aware profiling, human expert review, research-oriented output, and longitudinal updating.

### Clarifying the role of AI in a human-supervised architecture

The human-supervised nature of FIAP^®^-Digital does not remove the need for AI. Rather, it defines the limits and function of AI within the architecture. AI is needed because the relevant information is multimodal, temporally distributed, incomplete, and interaction-dependent. Manual review alone may struggle to organize repeated biological, physiological, developmental, contextual, therapeutic-process, and longitudinal indicators into coherent construct-level representations.

In this architecture, AI is proposed as an interpretable computational support layer with six restricted functions: (1) organizing heterogeneous inputs by modality, source, timestamp, quality, and missingness; (2) harmonizing variables across records and sites; (3) mapping indicators to provisional FIAP^®^ constructs; (4) estimating construct-level profiles with uncertainty flags; (5) identifying longitudinal patterns and profile shifts; and (6) generating clinician-readable explanations of profile drivers, data gaps, and uncertainty. The system does not make autonomous diagnostic, prognostic, eligibility, or treatment decisions.

Human supervision is therefore not a substitute for AI but the governance layer that makes AI use proportionate and ethically bounded. Clinicians and qualified researchers remain responsible for interpretation, contextualization, contestability, and deciding whether an output is meaningful for research use. This division of labor is consistent with responsible and explainable AI principles in healthcare, where computational systems may support pattern organization and uncertainty-aware interpretation without replacing human judgment (Amann et al., 2020; Gunning et al., 2019; Kelly et al., 2019; Sadeghi et al., 2024).

ALGORITHM 1 FIAP^®^-Digital human-supervised profiling workflow.1. Collect multimodal indicators from available biological, physiological, developmental, contextual, therapeutic-process, and longitudinal sources.
2. Document the source, timestamp, context, quality, and missingness status of each data element.
3. Preprocess and harmonize indicators within each modality while preserving data provenance.
4. Map candidate indicators to FIAP^®^ constructs, including BBI, energetic capacity, adaptive neurodevelopmental window accessibility, therapeutic engagement accessibility, and neuroplastic accessibility.
5. Generate preliminary construct-level estimates or profiles using transparent, uncertainty-aware methods.
6. Calculate data-completeness indicators and uncertainty estimates for each construct.
7. Infer higher-order translational profile configurations, such as burden-dominant, reserve-constrained, timing-sensitive, engagement-vulnerable, or mixed profiles.
8. Generate clinician-readable explanations describing profile drivers, construct contributions, missing-data limitations, and uncertainty.
9. Submit outputs to clinician or qualified researcher review for contextual interpretation, contestability, and revision.
10. Update construct estimates and translational profiles longitudinally as new data become available.
11. Use outputs only for research stratification, hypothesis generation, feasibility testing, and construct validation until empirical validity and clinical utility are established.

## Illustrative prototype scenario

To make the proposed architecture more concrete, an early FIAP^®^-Digital research prototype could begin with a minimal dataset rather than a fully automated multimodal platform. For example, a micro-pilot prototype could collect structured caregiver-reported indicators of sleep, gastrointestinal symptoms, fatigue, pain or discomfort, sensory-physiological stress, and contextual demands; clinician-rated observations of regulation, attentional access, responsiveness to support, persistence, and generalization; intervention-session descriptors such as intensity, setting, support type, and task demands; and repeated longitudinal ratings of participation and response stability.

The prototype would not generate a diagnosis, treatment recommendation, eligibility decision, or autonomous prediction. Instead, it would map available indicators to provisional construct-level representations such as biological burden, energetic capacity, adaptive neurodevelopmental window accessibility, therapeutic engagement accessibility, and neuroplastic accessibility. Each construct estimate would be accompanied by a data-completeness indicator, missing-data flags, source-provenance notes, and uncertainty estimates. The output would be a clinician-readable research profile describing possible profile drivers, low-confidence domains, and longitudinal change patterns. Such a prototype would be used only to evaluate feasibility, construct coherence, inter-rater interpretability, uncertainty calibration, and the empirical relationship between provisional FIAP^®^ constructs and subsequent engagement or responsiveness outcomes.

## Translational profile-inference engine

Once the core FIAP^®^ constructs have been estimated, the next computational layer is the translational profile-inference engine. This engine moves from individual construct estimates to higher-order multidomain configurations that may be more useful for research interpretation than isolated variables.

Profiles should be inferred from construct interaction rather than from raw data directly. They should remain probabilistic rather than rigid classes, allowing the system to represent burden-dominant, reserve-constrained, timing-sensitive, engagement-vulnerable, plasticity-constrained, and mixed multidomain configurations (Lombardo et al., 2019; Molloy et al., 2022; Beversdorf et al., 2023).

The profile engine should remain dynamic and updateable, preserve hierarchy of influence, support mixed-profile visibility, and remain clinically transparent. Its quality should be judged not only by predictive metrics, but by construct coherence, interpretability, temporal sensitivity, uncertainty calibration, and usefulness for hypothesis testing. At this stage, such profiles should not be treated as validated clinical categories.

## Explainability and clinician-in-the-loop design

The usefulness of FIAP^®^-Digital depends not only on the quality of its construct estimates or profile inferences, but on whether those outputs remain understandable, reviewable, and usable within a responsible research workflow. Explainability and clinician-in-the-loop design are therefore core architectural requirements.

Explainability in FIAP^®^-Digital must be construct-centered rather than purely feature-centered. Clinicians should be able to inspect broad input domains, construct-level estimates, profile drivers, missing-data indicators, and uncertainty. The system should provide reason-giving outputs rather than labels alone. Clinician-in-the-loop design must preserve human interpretive authority, support contestability, and enable contextual reintegration of outputs into broader developmental reasoning (Amann et al., 2020; Sadeghi et al., 2024; Albahri et al., 2023).

This design logic also affects prototype evaluation. A technically sophisticated system that improves some downstream metric but cannot be meaningfully reviewed by clinicians would not meet the requirements of the FIAP^®^ ecosystem. Interpretability, uncertainty awareness, and human supervision should therefore be evaluated alongside predictive or clustering performance.

## Prototype validation pipeline and implementation constraints

A credible FIAP^®^-Digital system requires a disciplined prototype validation pipeline. This pipeline should ensure that the system is tested first for input-layer reliability, then for construct fidelity, then for profile coherence, uncertainty calibration, interpretability, contestability, longitudinal change sensitivity, intervention-linked relevance, cross-context robustness, and only later for broader implementation value (Kelly et al., 2019; Topol, 2019; Albahri et al., 2023; Sohn et al., 2025).

Because FIAP^®^-Digital is not yet empirically validated, its scientific value depends on a staged validation roadmap. Table 3 summarizes the minimum validation domains required before any clinical utility or implementation claims could be considered.

**Table 3:** Validation roadmap for FIAP^®^-Digital.

Validation domain	Core question	Candidate methods	Success indicators	Key limitations to monitor
Feasibility validation	Can required data be collected with acceptable burden?	Micro-pilot testing; completion rates; time-to-completion; missing-data review; caregiver/clinician feedback	High completion of core variables; acceptable workflow burden; ability to generate preliminary profiles	Excessive questionnaire burden; missing key modalities; low usability
Construct validity	Do FIAP^®^ constructs measure the intended conceptual domains?	Expert review; content validity mapping; convergent and discriminant analyses; construct refinement; factor or latent structure exploration when sample size permits	Clear domain definitions; coherent item-to-construct mapping; evidence of construct separation and meaningful overlap	Construct overlap; poorly defined indicators; premature scoring
Internal validation	Are construct estimates and profiles coherent within the initial sample?	Cross-validation; sensitivity analyses; profile stability testing; internal consistency where appropriate	Stable profiles under reasonable assumptions; interpretable construct patterns; reproducible within-sample outputs	Overfitting; unstable profiles; dependence on small sample artifacts
External validation	Do profiles generalize across independent samples or sites?	Replication across cohorts, clinics, schools, or research settings; cross-site comparison	Comparable construct behavior across settings; generalizable profile logic	Site-specific bias; inconsistent data collection; population differences
Longitudinal validation	Do construct estimates track meaningful change over time?	Repeated measures; trajectory analysis; state-sensitive modeling; pre/post contextual changes	Profiles update coherently; change estimates correspond to observed clinical or functional changes	Regression to mean; inconsistent follow-up; temporal confounding
Uncertainty calibration	Are confidence estimates reliable and interpretable?	Calibration curves; confidence interval evaluation; probabilistic calibration; uncertainty auditing	Low-confidence outputs correspond to incomplete, noisy, or conflicting data; uncertainty is clinically visible	Overconfident outputs; hidden uncertainty; misleading precision
Clinician usability	Can clinicians understand, review, and contest outputs?	Usability testing; structured clinician feedback; think-aloud protocols; interpretability ratings	Clinicians can identify drivers, uncertainty, and limitations; outputs support review rather than automation	Cognitive burden; unclear explanations; false sense of authority
Fairness and bias assessment	Do outputs perform equitably across populations and settings?	Subgroup analyses by age, sex, language, socioeconomic context, race/ethnicity where appropriate, service access, and data availability	No systematic disadvantage due to missingness, access, demographic factors, or data quality	Dataset bias; digital divide; cultural or language bias; inequitable service implications
Low-resource adaptation	Can the architecture function with limited data availability?	Minimal dataset testing; tiered model versions; missingness-tolerant profiles; low-burden workflows	Usable low-data profile with transparent uncertainty; scalable implementation pathway	Exclusion of under-resourced families; excessive reliance on advanced sensors or biomarkers
Prospective pilot validation	Can FIAP^®^-Digital support future empirical hypothesis testing?	Prospective micro-pilot and macro-pilot studies; predefined hypotheses; preregistered outcomes where appropriate	Evidence that profiles can be generated, reviewed, and tested against engagement and responsiveness outcomes	Premature clinical translation; insufficient sample size; weak outcome linkage

Several implementation constraints must also be acknowledged: data richness and quality, construct immaturity, clinical workflow burden, heterogeneity of settings, ethical risk under premature scaling, overfitting to available modalities, and communication complexity. These constraints reinforce the broader message of the paper: FIAP^®^-Digital should advance as a carefully staged translational prototype, not as a rushed technology product.

## Implementation challenges and low-resource adaptation

The implementation of FIAP^®^-Digital would face several practical challenges that must be addressed before any prototype or clinical translation can be considered. The first challenge concerns multimodal data acquisition. Autism-related data are often distributed across families, clinicians, schools, intervention providers, medical records, and research settings. Biological, physiological, developmental, contextual, and therapeutic-process indicators may not be collected routinely, may be recorded in incompatible formats, or may vary in reliability across settings.

A second challenge concerns missingness. Missing data are expected to be common in autism research because of uneven access to services, family burden, sensory intolerance, cost, geographic location, institutional capacity, and differences in clinical documentation. For this reason, FIAP^®^-Digital should not assume complete multimodal data availability. Instead, it should begin with low-burden, clinically accessible indicators and explicitly represent missingness and uncertainty.

A third challenge is data harmonization. Different sites may use different assessment instruments, terminology, observation practices, intervention records, and data systems. Harmonization will therefore require structured variable definitions, data dictionaries, source documentation, and transparent mapping between observed indicators and FIAP^®^ constructs. Without such harmonization, apparent differences between profiles may reflect measurement differences rather than meaningful biological or functional variation.

Sensor variability and technological heterogeneity represent additional challenges. If future FIAP^®^-Digital prototypes incorporate wearable sensors, video/audio analysis, physiological monitoring, or biomarker data, the system will need to account for device differences, sampling frequency, signal quality, calibration, environmental noise, and participant tolerance. These issues are especially important in autism, where sensory sensitivities and behavioral variability may affect data capture.

Low-resource adaptation is central to the responsible development of FIAP^®^-Digital. A system that depends exclusively on advanced sensors, expensive biomarkers, or specialized neurotechnology could amplify inequities by excluding families and settings with limited access to technology or specialized care. For this reason, FIAP^®^-Digital should be developed as a tiered architecture. The first tier should rely on clinically accessible information, caregiver report, clinician observation, developmental-contextual variables, and structured therapeutic-process indicators. More advanced modalities, such as biomarkers, physiological sensors, video/audio analysis, or central neurodevelopmental measures, should be added only when feasible, ethically justified, and analytically interpretable.

Implementation must also consider workflow burden. Clinicians and families cannot be expected to complete excessively long forms or interpret complex algorithmic outputs. Any future prototype must therefore be evaluated for usability, time burden, interpretability, and perceived usefulness. A technically sophisticated system that cannot be integrated into real-world research or clinical workflows would not meet the goals of FIAP^®^-Digital.

Finally, implementation should proceed through staged testing: feasibility studies, micro-pilots, construct validation, prototype testing, longitudinal evaluation, cross-site replication, fairness assessment, and only later clinical translation. This staged approach is necessary to prevent premature scaling and to ensure that FIAP^®^-Digital remains scientifically valid, ethically responsible, and practically usable.

## Responsible AI, ethics, and governance

Responsible AI is not an optional add-on to FIAP^®^-Digital; it is a core design requirement. Autism-related digital systems may influence interpretation of developmental needs, intervention responsiveness, service planning, and family understanding. For this reason, any AI-supported architecture must be governed by transparency, interpretability, privacy protection, fairness, human supervision, and clear limits on use.

The first governance principle is human oversight. FIAP^®^-Digital should not generate autonomous diagnostic, prognostic, treatment, or service-allocation decisions. Outputs should be reviewed by clinicians or qualified researchers who can contextualize findings, consider alternative explanations, and identify limitations. Human review must include the ability to contest, revise, or reject algorithmic interpretations.

The second principle is interpretability. Outputs should be understandable at the construct level. Users should be able to see which domains contributed to BBI, energetic capacity, adaptive window accessibility, therapeutic engagement accessibility, neuroplastic accessibility, and higher-order profile configurations. The system should provide clinician-readable explanations, uncertainty flags, data-quality indicators, and missingness summaries.

The third principle is data minimization and privacy protection. FIAP^®^-Digital should collect only data that are necessary for the research question or validation stage. Identifying information should be separated from analytical data where possible, and participant-level data should be stored securely with access restricted to authorized personnel. Identifiable participant data should not be entered into external AI systems. All data use should comply with applicable ethical, institutional, legal, and consent requirements.

The fourth principle is transparency and documentation. Future prototypes should include data dictionaries, model documentation, version control, audit trails, and, where appropriate, model cards or datasheets describing intended use, limitations, training data, validation status, uncertainty behavior, and known risks. Such documentation is necessary to avoid misleading claims and to support reproducibility.

The fifth principle is fairness and bias assessment. FIAP^®^-Digital must be evaluated for differential performance across age, sex, language, socioeconomic context, race/ethnicity where appropriate, service access, geographic location, data availability, and communication profile. In autism research, bias may arise not only from demographic imbalance but also from differences in diagnostic access, intervention exposure, caregiver reporting, school support, and availability of biological or digital data.

The sixth principle is proportionality. The level of computational complexity should be appropriate to the stage of validation. Early FIAP^®^-Digital development should prioritize transparency, construct clarity, and feasibility rather than high-complexity modeling without sufficient data. More advanced AI methods should be introduced only when justified by sample size, data quality, validation maturity, and interpretability safeguards.

Together, these governance principles position FIAP^®^-Digital as a Responsible AI-oriented research architecture. Its purpose is to support empirical validation and hypothesis generation, not to replace clinical judgment or impose deterministic labels on autistic individuals.

## Integration within the proposed architecture

Figures 1, 2 are intended to serve complementary functions within the manuscript. Figure 1 summarizes the conceptual neuroinformatics architecture by showing how multimodal input layers are transformed into construct-level estimates and then into higher-order translational profiles. This directly reflects the manuscript’s distinction between prediction-centered AI systems and construct-preserving architectures: the figure makes visible the intermediate inferential layers that would otherwise be hidden in a purely black-box pipeline.

Figure 2 operationalizes the same logic as a human-supervised multimodal data-flow workflow. It translates the architectural model into an ordered process showing how biological, physiological, developmental, contextual, therapeutic-process, and longitudinal inputs move through provenance documentation, quality control, modality-specific harmonization, construct mapping, uncertainty-aware construct estimation, explainability, translational profile inference, human expert review, research-oriented output generation, and longitudinal updating. The revised figure also clarifies the division of labor between AI and human oversight: AI supports data organization, integration, construct estimation, uncertainty flagging, and explanation, whereas clinicians or qualified researchers retain responsibility for contextual interpretation, contestability, and approval for research use. Together, the two figures connect the literature on autism heterogeneity, multimodal AI, explainability, clinician oversight, and Responsible AI with a concrete sequence of research-development steps. Their purpose is not to demonstrate a validated implementation, but to specify how future FIAP^®^-Digital prototypes could be designed, audited, and empirically tested.

## Limitations and boundaries of interpretation

This manuscript has several important limitations. First, it is a conceptual Hypothesis and Theory article. It does not report original empirical data, prototype testing, computational experiments, statistical validation, or clinical trial findings. The architecture described should therefore be interpreted as a proposed research framework rather than as a validated digital health system.

Second, the core FIAP^®^ constructs described in this paper remain hypothesis-generating. The Biological Burden Index, energetic capacity, adaptive neurodevelopmental window accessibility, therapeutic engagement accessibility, neuroplastic accessibility, and translational profile configurations have not yet undergone formal construct validation. Their definitions, indicators, computational representations, and boundaries require empirical testing and refinement.

Third, no clinical evidence of effectiveness is claimed. FIAP^®^-Digital has not been shown to improve diagnosis, treatment selection, intervention outcomes, clinical decision-making, family support, or service allocation. Any future clinical relevance remains provisional and dependent on feasibility testing, construct validation, prospective evaluation, external replication, clinician usability assessment, fairness analysis, and ethical review.

Fourth, the proposed computational architecture is not an implemented algorithm. The manuscript outlines candidate methods for multimodal fusion, temporal modeling, uncertainty estimation, explainability, and human-supervised interpretation, but it does not present an operational software platform or validated model. These methodological components should be viewed as a roadmap for future development.

Fifth, the manuscript does not resolve the challenge of autism heterogeneity. Instead, it proposes a structured approach for testing whether multidomain biological burden, adaptive reserve, developmental timing, therapeutic engagement, and neuroplastic accessibility can be represented computationally in ways that are meaningful for future research. It is possible that some FIAP^®^ constructs will require revision, subdivision, or replacement after empirical testing.

Sixth, implementation may be limited by data availability, missingness, measurement variability, site differences, resource constraints, and participant burden. A responsible FIAP^®^-Digital pathway must therefore begin with low-burden research use and avoid premature clinical deployment.

Finally, FIAP^®^-Digital should not be used as an autonomous diagnostic, predictive, treatment recommendation, or clinical decision-making system. Its current value lies in defining a research architecture and validation pathway for future precision-stratified autism research.

## Conclusion

FIAP^®^-Digital is proposed as a conceptual, hypothesis-generating, and human-supervised multimodal AI architecture for future autism stratification research. Its central contribution is to define how multimodal biological, physiological, developmental, contextual, therapeutic-process, and longitudinal indicators could be organized into construct-level representations and higher-order translational profiles while preserving interpretability, uncertainty visibility, and human oversight.

The revised architecture emphasizes that autism translational heterogeneity cannot be reduced to diagnostic classification or black-box prediction alone. Instead, future research may require construct-preserving computational approaches capable of testing relationships among biological burden, energetic capacity, developmental timing, therapeutic engagement, neuroplastic accessibility, and intervention responsiveness.

At this stage, FIAP^®^-Digital should not be interpreted as a validated clinical tool, evidence-based intervention platform, or implemented software product. The FIAP^®^ constructs remain hypothetical, and their construct validity, predictive relevance, clinical utility, fairness, usability, and longitudinal robustness require empirical evaluation. The appropriate next steps include feasibility testing, operational refinement, minimal prototype development, construct validation, internal and external validation, uncertainty calibration, Responsible AI assessment, and prospective pilot studies.

By articulating this architecture and validation roadmap, the manuscript provides a neuroinformatics foundation for future empirical testing of FIAP^®^ constructs. If validated, such a framework may eventually contribute to precision-stratified autism research and more responsible, interpretable, and developmentally sensitive digital phenotyping approaches.

## Data Availability

The original contributions presented in the study are included in the article/supplementary material, further inquiries can be directed to the corresponding author.
